# Patterns of Professional Practice Participation: A Latent Profile Analysis of Specialty Nurses in China

**DOI:** 10.1155/jonm/5574431

**Published:** 2026-01-22

**Authors:** Yan Ye, Yinshi Kan, Tingting Dong, Jing Zhang, Xiaoming Li, Lingyu Zhang, Xuanxuan Fan, Chunyan Li, Liwei Jing

**Affiliations:** ^1^ School of Nursing, Capital Medical University, Beijing, China, ccmu.edu.cn; ^2^ Department of Nursing, Xuanwu Hospital Capital Medical University, Beijing, China, xwhosp.com.cn; ^3^ School of Public Health, Harbin Medical University, Harbin, China, hrbmu.edu.cn; ^4^ School of Public Health, North China University of Science and Technology, Tangshan, China, ncst.edu.cn; ^5^ Beijing Nursing Association, Beijing, China

**Keywords:** latent profile analysis, participation, professional development, specialty nurses

## Abstract

**Background:**

Specialty nurses play a vital role in advancing clinical care, education, research, and leadership within China’s evolving healthcare system. However, their actual participation in these roles varies widely, and current assessments often overlook individual differences. Understanding distinct patterns of specialty nurses’ professional practice participation is crucial for optimizing talent development and advancing the specialty nursing workforce.

**Methods:**

A cross‐sectional study was conducted using a convenience sample of 7154 specialty nurses in Beijing between May and July 2024. Participants completed online questionnaires that gathered demographic data and assessed nursing practice across four dimensions (19 items total). Data analysis was performed using latent profile analysis, Chi‐square test, Kruskal–Wallis H test, and multivariate logistic regression.

**Results:**

Three latent profiles were identified: Developing Practice Profile (78.19%; characterized by growth potential across all dimensions), Clinician–Educator Profile (14.97%; marked by strengths in teaching and advanced clinical nursing), and the Clinician, Leadership, and Research Profile (6.84%; distinguished by high performance in advanced clinical practice, clinical management, and nursing research). Key predictors included educational background, professional title, duties, years of relevant work experience, and hospital level.

**Conclusion:**

Most specialty nurses have significant developmental potential. Nursing managers should provide personalized development for each specialty nurse, and the proposed “Stretch–Shape–Lead” approach may serve as a reference for optimizing the specialty nurse workforce structure. Future studies could further validate the findings from this resource‐rich municipality in lower‐tier cities, rural areas, or the broader national context to confirm their broader applicability.

**Reporting Method**

• We adhered to the STROBE guideline (reported in Supporting File 1).

## 1. Introduction

Nurses represent the largest segment of the professional healthcare workforce and play a crucial role in achieving global health coverage, particularly in addressing the needs of aging populations [1]. Globally, advanced practice nursing roles, such as nurse practitioners and clinical nurse specialists (CNSs), are becoming increasingly important [2, 3]. In China, the demand for nurses with advanced skills is also growing, although a formal advanced practice nursing system is still in development.

In the Chinese context, the role of CNS is often referred to as specialty nurse [2]. Specialty nurse serves as the primary pathway for developing advanced nursing expertise in the country and form the foundation of broader advanced nursing practice. Most experts agree that a specialty nurse is a registered nurse who has received structured training in nursing practice and theoretical knowledge within a specific nursing specialty. In China, specialty nurses are also required to pass a formal evaluation and obtain certification from the Medical and Health Administration [2]. The *National Nursing Development Plan (2021–2025)* [4] emphasizes the need to expand nursing specialty alliances and enhance training in high‐demand specialties, reinforcing the critical role of specialty nurse workforce development in China.

Since 2003, Beijing has led the way in specialty nurse training through its Intensive Care Specialty Nurse Training Class, setting an example for the rest of mainland China [5]. As a national medical center, Beijing has a high demand for nursing services [6], compounded by a rapidly aging population, which is 1.5% points higher than the national average [7]. These factors necessitate advanced nursing competencies among specialty nurses, making Beijing an ideal setting for examining specialty nurse practices.

Specialist practice encompasses clinical, teaching, administration, research, and consultant roles [8]. In China, specialty nurses are expected to manage complex clinical problems, serve as educators and coordinators, conduct research, and assume leadership responsibilities [2, 9], all of which contribute to significantly improving care quality and patient outcomes [10, 11]. While these expectations align with broader advanced practice nursing competencies, the actual nursing practice varies among specialty nurses [12, 13]. However, previous studies have examined specialty nurse’ practices using total scale score or the average score of each dimension, such as clinical care, teaching, management, and research [14, 15], without taking into account individual differences and potential subgroup variations. For instance, Li et al. [16] assessed diabetes specialty nurses’ health education practices using the *Nurse Health Education Competency Scale (I-CepSE)*, while Unsworth et al. [17] introduced the Family and Community Nursing Advanced Practice Scale to measure practice scope and frequency. Tools, such as the *Advanced Practice Role Delineation tool* [18–20], also allow for comparison of practice levels across five domains: direct care, system support, education, research, and leadership.

Theoretical views on nursing development can explain how nurses acquire expertise and evolve in their practice over time. Benner’s [21] *From Novice to Expert* model frames clinical skill development as a progression through experiential stage. This core principle—that experience fundamentally reshapes practice—is particularly applicable to the complex roles of specialty nurses. It suggests that, over time, specialty nurses may manifest distinct patterns in their practice, reflecting different combinations of engagement across clinical, teaching, management, and research dimensions.

Latent profile analysis is a person‐centered statistical method that identifies distinct subgroups based on observed variable scores, with applications in areas such as personal characteristics and career interventions [22]. Latent profile analysis [23, 24] complements this framework by probabilistically classifying individuals into subgroups based on multidimensional practice scores, revealing distinct patterns of practice engagements. Recognizing that some specialty nurses focus more on education, while others emphasize research, this study aimed to (a) explore the potential nursing practice profile types of specialty nurses in Beijing and (b) use a series of demographic and nursing practice–related variables to identify the predictors of each profile.

## 2. Methods

### 2.1. Study Design

A cross‐sectional study with latent profile analysis was carried out.

### 2.2. Sampling Strategy

A convenience sampling approach was used to recruit specialty nurses from all public hospitals in Beijing between May and July 2024. Eligibility criteria include the following: (i) holding a Chinese nursing practice qualification certificate within its valid registration period; (ii) being certified as a specialty nurse by the Beijing Nursing Association in the relevant field.

### 2.3. Sample Size

The sample size was estimated based on the common guideline of using 10 to 15 times the number of questionnaire items [25, 26]. Given that there are 19 items in this questionnaire, the baseline sample size was calculated as *N* = 19 ∗ 15 = 285, which means that at least 190 subjects are needed for this study. To account for potential low‐quality or invalid responses, it is advisable to adjust the sample size accordingly. Therefore, the minimum sample size required is *N* = 285 ÷ (1%–20%) ≈ 356. The revised sample size aligns with recommendations from Nylund‐Gibson and Choi [27], who suggest that the minimum sample size for latent profile analysis should be 300.

### 2.4. Data Collection

This study was carried out using an online questionnaire survey distributed through an online data collection website named “Wenjuanxing”. The questionnaire links were distributed using a convenience sampling method. The Beijing Nursing Association facilitated access to the Nursing Department of hospitals at the first, second, and third levels in the 16 districts of Beijing. The Nursing Department then sent the links of the online questionnaire to the specialty nurses in their respective hospitals. Participation was anonymous and voluntary with informed written consent obtained from respondents before completing the questionnaire.

### 2.5. Instruments

#### 2.5.1. Demographic Characteristics

The demographic data collected from participants in this study include the following 11 items: age, gender, name of institution, type and level of hospital, years of nursing experience and obtaining the specialty certification, years of working experience in related fields, educational background, professional title, and duties.

#### 2.5.2. Questionnaire on the Professional Practice Participation of Specialty Nurses in Beijing

This study assessed the current state of the professional practice of specialty nurses in Beijing across four dimensions: advanced clinical nursing, nursing management, nursing teaching, and nursing research. With prior permission from the original authors, two previously published questionnaires were adapted as primary references: Ding et al. [14] and Zhao et al. [15]. A comprehensive review of relevant domestic and international literature on specialty nursing was also conducted to inform the questionnaire design. An initial draft of the research questionnaire was developed and subsequently refined through multiple rounds of expert panel discussions. The panel comprised three nursing department directors, four senior specialty nurses from diverse departments and hospital levels, four specialized nursing professors, and two statistical experts. After iterative revisions, the final version, titled *“Questionnaire on the Professional Practice Participation of Specialty Nurses in Beijing”* was established. The questionnaire employed a 4‐point Likert scale (1–4 points, ranging from *“low participation”* to *“high participation”*) to evaluate specialty nurses’ engagement in each of the 19 items. The total score ranged from 19 to 76, with higher scores indicating greater participation levels. The scale demonstrated good internal consistency, with a Cronbach’s *α* coefficient of 0.818.

### 2.6. Data Analysis

To ensure data quality and facilitate subsequent statistical analysis, questionnaires with a response time of less than 300 s (*N* = 513) were deleted, and the missing values were imputed using the median. In addition, questionnaires with all contribution amounts filled as “0” (*N* = 162) were deleted, as these may indicate that the respondents did not fill them out carefully.

This study used Mplus 8.3 and SPSS 27.0 software for data processing. The Mplus 8.3 software was used to conduct a latent profile analysis on the types of roles played by specialty nurses in Beijing. The process started from a single‐class model and gradually increased the number of classes to determine the model that best fit the data, which were determined as the final model. The model fit indices included the Akaike information criterion (*AIC*), Bayesian information criterion (*BIC*), adjusted BIC (*aBIC*), Lo–Mendell–Rubin adjusted likelihood ratio test (*LMRT*), bootstrapped likelihood ratio test (*BLRT*), entropy, and class counts and proportions. The criteria for determining the optimal model included the following: *AIC*, *BIC*, and *aBIC* being smaller than those of other competing models; the entropy index being greater than 0.7 (closer to 1); and the significance of the *LMRT* and *BLRT* with *p* < 0.05 [28–30]. *LMRT* and *BLRT* were used to compare the fit differences between the *k*‐class model and the *k*−1 class model [30]. Class sizes were also evaluated to ensure that each profile comprised at least 5% of the total sample [29]. SPSS 27.0 statistical software was used for data analysis. Categorical variables were presented as frequencies and percentages (%). The Chi‐square test and Kruskal–Wallis *H* test were used to assess group differences for the intergroup comparisons of unordered and ordered categorical variables, respectively. Multinomial logistic regression was conducted, with univariate analysis–selected variables as independent variables and distinct professional practice participation profiles as dependent variables, and a test level of *α* = 0.05. The variance inflation factor (VIF) was used to assess multicollinearity among predictors for the logistic regression model. VIF values above 5–10 indicate the presence of multicollinearity [31].

### 2.7. Ethical Considerations

This study has been reviewed and approved by the Ethics Review Committee of Capital Medical University (Ethics Approval Number: Z2024SY040).

## 3. Results

### 3.1. Sample Characteristics

A total of 7154 nurses participated and completed this study yielding a response rate of 91.38%. The average age of the participants was 38.19 years old (*SD* = 6.108), with most of them being female comprising 96.7% of the sample. Most respondents worked in tertiary hospitals (92.2%), with 58.6% having been certified as specialized nurses 5 years or fewer. Among them, 45.8% had 11–20 years of working experience in relevant specialties, 89.7% had a bachelor’s degree, 66.5% held the professional title of charge nurse, and 44.4% had the responsibility or position of primary nurse. Table 1 provides an overview of the demographic characteristics of the participants.

**Table 1:** Demographic characteristics and professional practice patterns in different latent profiles (*N* = 7154).

Characteristic	Overall (*N = *7154)	C1 (*n* = 5594) *n* (%)	C2 (*n* = 1071) *n* (%)	C3 (*n* = 489) *n* (%)	Test statistics	*p*
Age					80.034^2^	**<** **0.001**
≤ 30	613 (8.6)	556 (9.9)	42 (3.9)	15 (3.1)		
31–40	4231 (59.1)	3343 (59.8)	622 (58.1)	266 (54.4)		
41–50	1965 (27.5)	1445 (25.8)	351 (32.8)	169 (34.6)		
≥ 51	345 (4.8)	250 (4.5)	56 (5.2)	39 (8.0)		
Gender					1.308^1^	0.520
Male	235 (3.3)	177 (3.2)	41 (3.8)	17 (3.5)		
Female	6919 (96.7)	5417 (96.8)	1030 (96.2)	472 (96.5)		
Type of hospital					3.979^1^	0.137
General hospital	5858 (81.9)	4606 (82.3)	855 (79.8)	397 (81.2)		
Specialized hospital	1296 (18.1)	988 (17.7)	216 (20.2)	92 (18.8)		
Level of hospital					66.089^2^	**<** **0.001**
Nontertiary hospitals	561 (7.8)	513 (9.2)	42 (3.9)	6 (1.2)		
Tertiary hospital	6593 (92.2)	5081 (90.8)	1029 (96.1)	483 (98.8)		
Years of nursing experience					62.524^2^	**<** **0.001**
≤ 10	1332 (18.6)	1142 (20.4)	133 (12.4)	57 (11.7)		
11–20	4086 (57.1)	3171 (56.7)	644 (60.1)	271 (55.4)		
≥ 21	1736 (24.3)	1281 (22.9)	294 (27.5)	161 (32.9)		
Years of obtaining the specialty qualification					66.358^2^	**<** **0.001**
≤ 5	4191 (58.6)	3408 (60.9)	562 (52.5)	221 (45.2)		
6–10	2099 (29.3)	1558 (27.9)	361 (33.7)	180 (36.8)		
11–15	633 (8.8)	456 (8.2)	116 (10.8)	61 (12.5)		
≥ 16	231 (3.2)	172 (3.1)	32 (3.0)	27 (5.5)		
Years of work experience in relevant fields					87.345^2^	**<** **0.001**
≤ 10	3051 (42.6)	2538 (45.4)	359 (33.5)	154 (31.5)		
11–20	3256 (45.8)	2455 (43.9)	556 (51.9)	245 (50.1)		
≥ 21	847 (11.8)	601 (10.7)	156 (14.6)	90 (18.4)		
Educational background					151.744^2^	**<** **0.001**
Associate degree and below	590 (8.2)	531 (9.5)	50 (4.7)	9 (1.8)		
Bachelor’s degree	6417 (89.7)	5000 (89.4)	988 (92.3)	429 (87.7)		
Master’s degree and above	147 (2.1)	63 (1.1)	33 (3.1)	51 (10.4)		
Professional title					369.961^2^	**<** **0.001**
Senior nurse and below	1975 (27.6)	1765 (31.6)	172 (16.1)	38 (7.8)		
Supervisor nurse	4758 (66.5)	3627 (64.8)	792 (73.9)	339 (69.3)		
Associate chief nurse	389 (5.4)	188 (3.4)	98 (9.2)	103 (21.1)		
Chief nurse	32 (0.4)	14 (0.3)	9 (0.8)	9 (1.8)		
Duties						
Primary nurse	3174 (44.4)	2774 (49.6)	303 (28.3)	97 (19.8)	605.015^1^	**<** **0.001**
Responsible team leader	1642 (23.0)	1191 (21.3)	362 (33.8)	89 (18.2)		
Head nurse	1320 (18.5)	836 (14.9)	271 (25.3)	213 (43.6)		
Head nurse of department	139 (1.9)	70 (1.3)	35 (3.3)	34 (7.0)		
Director and deputy director of nursing department	47 (0.7)	22 (0.4)	12 (1.1)	13 (2.7)		
Others	832 (11.6)	701 (12.5)	88 (8.2)	43 (8.8)		

*Note:* C1 = Developing Practice Profile; C2 = Clinician–Educator Profile; C3 = Clinician, Leadership, and Research Profile; M, mean. Bold numbers represent *p* < 0.05.

Abbreviation: SD, standard deviation.

^1^Chi‐squared value.

^2^ *H* value.

### 3.2. Latent Profiles of Professional Practice Participation Among Specialty Nurses in Beijing

In this study, five latent profile models were fitted, and the fitting indices of each model are shown in Table 2. The *AIC*, *BIC*, and *aBIC* gradually decreased as the number of classes increased. The entropy values of all profiles were greater than 0.9, and the *LMRT* and *BLRT* were statistically significant (*p* < 0.05). However, the sizes in 4‐profile and 5‐profile models represented less than 5% of the total, making the findings difficult to interpret. Therefore, the 3‐profile model was considered the best‐fitting solution for the following reasons: (a) it had lower *AIC* (319,088.279)*, BIC* (319,624.562), and *aBIC* (319,376.696) values than the 2‐profile model; (b) the entropy value (0.987) was higher, and the size in the smallest subtype was 6.8%; and (c) the *LMRT* (*p* < 0.001) and *BLRT* (*p* < 0.001) were significant at *α* = 0.05.

**Table 2:** Fit indices of each model.

Model	AIC	BIC	aBIC	*p* value		Entropy	Class counts and proportions
				LMRT	BLRT		
One‐profile	349,081.167	349,342.950	349,222.194	—	—	—	—
Two‐profile	329,629.394	330,028.958	329,844.646	< 0.001	< 0.001	1.000	0.067/0.933
Three‐profile	319,088.279	319,624.562	319,376.696	< 0.001	< 0.001	0.987	0.782/0.150/0.068
Four‐profile	312,486.914	313,160.706	312,849.284	0.001	< 0.001	0.967	0.050/0.164/0.752/0.034
Five‐profile	306,971.400	307,782.701	307,407.723	0.026	< 0.001	0.972	0.075/0.698/0.146/0.034/0.048

*Note:* LMRT: Lo–Mendell–Rubin likelihood ratio test.

Abbreviations: aBIC = adjusted Bayesian information criterion, AIC = Akaike information criterion, BIC = Bayesian information criterion, and BLRT = bootstrapped likelihood ratio test.

**Figure 1:**
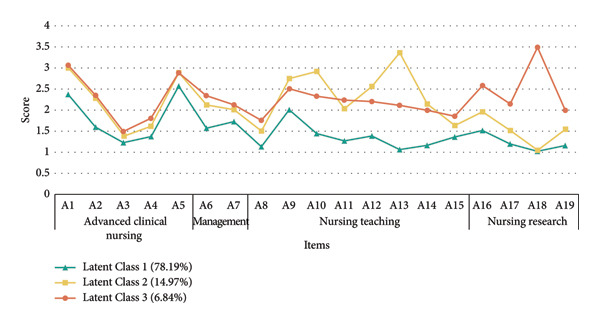
Latent profiles of professional practical participation among the specialty nurses in Beijing. *Notes*: The different participation of professional practice among specialty nurses. Class 1: Developing Practice profile; Class 2: Clinician–Educator Profile; Class 3: Clinician, Leadership, and Research Profile.

The scores of the three classes on the 19 questionnaire items are presented in Figure 1.

Profile 1 had the lowest scores across all dimensions of specialty nursing practice, suggesting limited participation in specialty nursing practices. We named this group the “*Developing Practice Profile*” to reflect nurses who are still in the early stages of developing their specialty practice. This group accounted for 78.19% of the sample (*n* = 5594). Profile 2 showed a high score in the advanced clinical nursing dimension, slightly lower than that of Profile 3. Meanwhile, they scored highest in nursing education activities, including clinical teaching and theoretical teaching volume for nursing students and in‐service training personnel, the cultivation of specialty nurses. We named this group the “*Clinician–Educator Profile.*” It comprised 14.97% of the sample (*n* = 1071). Profile 3 was distinguished by consistently high scores in advanced clinical nursing (e.g., managing complex or critical cases, clinics, and consultations), clinical nursing management (e.g., improving quality and patient care), and nursing research. They could integrate clinical expertise, leadership in clinical nursing, and scholarly activities. We named this group the “*Clinician, Leadership*, *and Research Profile.*” This group represented 6.84% of the sample (*n* = 489).

### 3.3. Demographic and Work‐Related Characteristics of Each Profile

The median and interquartile range of the questionnaires of all specialty nurses in Profiles 1, 2, and 3 were 27 (24.31), 39 (35.45), and 43 (35.50), respectively, which are shown in Table 3. Table 1 displays the frequency and percentage of demographic characteristics and professional practice participation status for each profile.

**Table 3:** Scores on the four dimensions of professional practice participation across three profiles (*N *= 7154), M (P_25_,P_75_).

Dimensions	Total sample (*N* = 7154)	C1 (*n* = 5594)	C2 (*n* = 1071)	C3 (*n* = 489)	*H*	*p*
Advanced clinical nursing	8 (6.10)	7 (6.9)	10 (8.12)	10 (8.13)	685.806	< 0.001
Nursing management	3 (2.5)	3 (2.4)	4 (2.5)	4 (3.6)	375.218	< 0.001
Nursing teaching	10 (7.13)	9 (7.11)	17 (15.19)	15 (11.19)	2672.102	< 0.001
Nursing research	7 (6.9)	7 (6.8)	8 (7.10)	13 (11.15)	1446.357	< 0.001
Total	29 (25.35)	27 (24.31)	39 (35.45)	43 (35.50)	2443.650	< 0.001

*Note:* C1 = Developing Practice Profile; C2 = Clinician–Educator Profile; C3 = Clinician, Leadership, and Research Profile; M (P25, P75): median and interquartile range.

Among specialty nurses in the “Developing Practice Profile”(C1), close to three‐fifths (59.8%) were aged 31–40 years, with most having less than or equal to 5 years of experience in obtaining the specialty nurse qualification (60.9%). Those with less than or equal to 10 years of experience in the relevant specialty field accounted for the largest proportion (45.4%).

In the “Clinician–Educator Profile”(C2), slightly above one‐third (33.7%) had obtained the specialty nurse qualification for 6–10 years, 33.8% served as responsible team leaders, nearly three‐quarters (73.9%) were supervisor nurses, and the largest proportion (60.1%) had 11–20 years of nursing experience.

In the “Clinician, Leadership, and Research Profile”(C3), 10.4% held a master’s degree or higher and 98.8% worked in tertiary hospitals, both of which were the highest proportions among the three groups. Notably, 43.6% were head nurses, and 22.9% (21.1% associate chief nurse + 1.8% chief nurse) held the title of associate chief nurse or above (see Table 1).

### 3.4. Predictor of Latent Profile Membership

The results of the multivariate logistic regression analysis are shown in Table 4, and predictors of latent profile membership were set in bold. The predictors included in the model have passed the collinearity test to ensure no multicollinearity issues, with detailed results presented in Supporting Table 1.

**Table 4:** The multifactor analysis of three latent profiles of the professional practice patterns of specialty nurses by logistic regression (*N* = 7154).

Characteristics	C1 VS C3				C2 VS C3				C2 VS C1			
	*β*	OR	95% CI	*p*	*β*	OR	95% CI	*p*	*β*	OR	95% CI	*p*
*Age*												
≤ 30	−0.389	0.678	(0.305, 1.504)	0.339	−0.523	0.593	(0.241, 1.456)	0.254	−0.134	0.875	(0.506, 1.511)	0.631
31–40	−0.385	0.680	(0.403, 1.150)	0.150	−0.302	0.739	(0.410, 1.335)	0.317	0.083	1.087	(0.726, 1.628)	0.686
41–50	−0.053	0.948	(0.616, 1.458)	0.808	0.102	1.108	(0.679, 1.808)	0.682	0.156	1.168	(0.826, 1.652)	0.379
≥ 51	0^b^	—	—	—	0^b^	—	—	—	0^b^	—	—	—

*Level of hospital*												
Nontertiary hospitals	2.270	9.677	(4.238, 22.099)	**<** **0.001**	1.390	4.013	(1.678, 9.598)	**0.002**	−0.880	0.415	(0.297, 0.579)	**<** **0.001**
Tertiary hospital	0^b^	—	—	—	0^b^	—	—	—	0^b^	—	—	—
Years of nursing experience												
≤ 10	−0.179	0.836	(0.535, 1.308)	0.433	−0.039	0.961	(0.584, 1.582)	0.877	0.139	1.149	(0.835, 1.582)	0.393
11–20	−0.143	0.867	(0.599, 1.254)	0.449	0.053	1.054	(0.700, 1.588)	0.801	0.195	1.216	(0.937, 1.577)	0.142
≥ 21	0^b^	—	—	—	0^b^	—	—	—	0^b^	—	—	—

*Years of obtaining the specialty qualification*												
≤ 5	0.148	1.159	(0.704, 1.910)	0.561	0.450	1.569	(0.874, 2.815)	0.131	0.302	1.353	(0.889, 2.059)	0.158
6–10	−0.142	0.868	(0.530, 1.422)	0.574	0.294	1.342	(0.752, 2.395)	0.319	0.436	1.546	(1.018, 2.349)	**0.041**
11–15	−0.002	0.998	(0.587, 1.696)	0.994	0.379	1.461	(0.788, 2.708)	0.229	0.381	1.464	(0.939, 2.280)	0.092
≥ 16	0^b^	—	—	—	0^b^	—	—	—	0^b^	—	—	—

*Years of work experience in relevant fields*												
≤ 10	0.500	1.649	(1.149, 2.365)	**0.007**	0.161	1.174	(0.783, 1.760)	0.437	−0.339	0.712	(0.547, 0.928)	**0.012**
11–20	0.280	1.323	(0.934, 1.874)	0.115	0.155	1.168	(0.790, 1.725)	0.437	−0.125	0.883	(0.681, 1.144)	0.345
≥ 21	0^b^	—	—	—	0^b^	—	—	—	0^b^	—	—	—

*Educational background*												
Associate degree and below	2.521	12.441	(5.529, 27.991)	**<** **0.001**	1.422	4.146	(1.723, 9.977)	**0.002**	−1.099	0.333	(0.192, 0.578)	**<** **0.001**
Bachelor’s degree	1.492	4.447	(2.861, 6.912)	**<** **0.001**	0.791	2.205	(1.357, 3.582)	**0.001**	−0.702	0.496	(0.314, 0.781)	**0.003**
Master’s degree and above	0^b^	—	—	—	0^b^	—	—	—	0^b^	—	—	—

*Professional title*												
Senior nurse and below	1.593	4.916	(1.768, 13.667)	**0.002**	0.530	1.698	(0.565, 5.101)	0.345	−1.063	0.345	(0.137, 0.868)	**0.024**
Supervisor nurse	0.637	1.891	(0.730, 4.897)	0.189	0.058	1.059	(0.382, 2.938)	0.912	−0.580	0.560	(0.228, 1.378)	0.207
Associate chief nurse	−0.202	0.817	(0.318, 2.100)	0.675	−0.292	0.747	(0.273, 2.043)	0.570	−0.090	0.914	(0.369, 2.262)	0.846
Chief nurse	0^b^	—	—	—	0^b^	—	—	—	0^b^	—	—	—
*Duties*												

Primary nurse	0.382	1.465	(1.002, 2.142)	**0.049**	0.376	1.456	(0.939, 2.258)	0.093	−0.006	0.994	(0.768, 1.286)	0.964
Responsible team leader	−0.202	0.817	(0.556, 1.200)	0.303	0.678	1.969	(1.271, 3.052)	**0.002**	0.880	2.411	(1.866, 3.114)	**<** **0.001**
Head nurse	−1.148	0.317	(0.222, 0.454)	**<** **0.001**	−0.362	0.696	(0.458, 1.057)	0.089	0.786	2.194	(1.676, 2.871)	**<** **0.001**
Head nurse of department	−1.616	0.199	(0.112, 0.352)	**<** **0.001**	−0.477	0.621	(0.327, 1.177)	0.144	1.140	3.126	(1.910, 5.115)	**<** **0.001**
Director and deputy director of nursing department	−1.929	0.145	(0.061, 0.346)	**<** **0.001**	−0.606	0.545	(0.215, 1.382)	0.201	1.323	3.754	(1.706, 8.262)	**0.001**
Others	0^b^	—	—	—	0^b^	—	—	—	0^b^	—	—	—

*Note:* C1 = Developing Practice Profile; C2 = Clinician–Educator Profile; C3 = Clinician, Leadership, and Research Profile; β, unstandardized coefficient; bold numbers represent *p* < 0.05.

Abbreviations: CI, confidence interval; OR, odds ratio.

We observed that, compared with the “Clinician, Leadership, and Research Profile”(C3), the “Developing Practice Profile”(C1) was more likely to consist of a primary nurse with limited experience (≤ 10 years), lower degrees (Associate/Bachelor’s), and lower professional titles (nurse or below).

Compared with the “Clinician, Leadership, and Research Profile”(C3), the “Clinician–Educator Profile”(C2) was more likely to be primary group leaders, held lower degrees (Associate/Bachelor’s), worked predominantly in first‐/second‐level hospitals, and were less likely to be head nurses or above.

Compared with the “Developing Practice Profile”(C1), the “Clinician–Educator Profile”(C2) was more likely to have obtained the qualification of specialty nurses for 6–10 years, hold positions of responsible team leader, head nurse or above. They were likely to have more than 10 years of working experience, hold Associate or Bachelor’s degrees, have professional titles of nurse or below, or work in first‐/second‐level hospitals.

## 4. Discussion

To the best of our knowledge, this is the first study on the practice types of specialty nurses in Beijing. For Objective a, our latent profile analysis identified that the professional practice participation of specialty nurses in Beijing can be divided into three characteristics: a group with greater potential for growth (Developing Practice Profile), a group with the highest levels of participation in teaching and good performance in advanced clinical nursing (Clinician–Educator Profile), and a group with the highest levels of participation in advanced clinical nursing, nursing management, and nursing research (Clinician, Leadership, and Research Profile). For Objective b, we have also observed differences in influencing factors among different profiles.

### 4.1. Implications of Plausible Tailored Interventions for the Three Profiles and Alignment With Benner’s Model

This study identified three distinct practice profiles among specialty nurses, aligning with Benner’s “From Novice to Expert” model [21]. Understanding the potential positions of these profiles on the professional development ladder can help us formulate more targeted development strategies [32].

Nurses in the Developing Practice Profile accounted for most of the sample (78.19%), and their scores on all items of the questionnaire were significantly lower than those of other groups. This suggests that most specialty nurses in this study may be in the early stages of developing broad professional competencies, forming a bottom‐heavy structure. This group corresponds to the Competent to early Proficient stages. Among the nurses in this group, a higher proportion had obtained the specialty nurse qualification for less than 5 years, had relevant professional working experience of less than 10 years, and held the position of a primary nurse. Their relatively shorter tenure as specialty nurses means their accumulation of advanced capabilities in clinical practice, management, education, and research suggesting they are in the early stages of evolving into well‐rounded professionals [33]. Crucially, while demonstrating lower engagement levels in advanced activities compared to other profiles, they possess formal specialist training and foundational skills, distinguishing them fundamentally from general registered nurses [26]. This group represents a significant pool of developing talent, an often under‐recognized and potentially underutilized resource. The heavy clinical workload of primary nurses also limits specialty nurses’ opportunities to apply their advanced specialty nursing competencies [34]. The “Stretch” strategy is proposed for this group. It is recommended to establish a hierarchical utilization mechanism for specialty nurses and a “clinical tutoring system” for those with less than 5 years of qualification and implement personalized development programs aligned with their goals, featuring increased rotations in advanced practice (e.g., complex cases, initial research, and teaching support).

Nurses in the Clinician–Educator Profile have the highest scores in clinical teaching for nursing students and in‐service training personnel, theoretical teaching, while performing well in advanced clinical nursing, showing focused development in specific directions. This group likely represents nurses operating at the proficient or even expert level. This group is mainly composed of nurses who have 11–20 years of relevant professional working experience and hold the position of a responsible team leader. Analyzing the reasons, it is likely that nurses with a longer working experience often accumulate richer clinical work experience and more in‐depth professional knowledge [35]. They can accurately point out the key points of operations and guide nursing students and training personnel to carry out more complex technical operations. During the teaching process, they can continuously reinforce theoretical and practical knowledge and improve their own clinical practice capabilities; hence, their teaching activities not only benefit others but also reinforce their own practice skills. In addition, role demands play a significant part. Responsible team leaders are typically tasked with coordinating team quality, safety, and training, naturally fostering stronger specialized capabilities and educational competencies [36]. Furthermore, Beijing’s concentration of major teaching hospitals attracts many nurses seeking advanced training, creating high demand for skilled clinical educators. It is recommended to “Shape” their expertise by enhancing their expertise in clinical teaching. This includes supporting them in developing teaching materials, participating in curriculum design, and undertaking higher‐level teaching responsibilities, as well as providing pathways for them to become certified clinical education mentors or specialty area education consultants. Additionally, according to their individual willingness and potential, opportunities to expand into management or research fields should be offered.

The Clinician, Leadership, and Research Profile accounts for the smallest proportion, at only 6.84%. It has the highest level of participation in advanced clinical nursing, nursing management, and nursing research, representing a category of specialty nurses with comprehensive participation and a high level of professionalism. This group aligns with the expert stage and often represents current or potential advanced practice nurses. Most of the nurses in this group typically have ≥ 11 years of experience and hold senior positions such as associate chief nurse or above. They possess richer specialty nursing experience, assume more management functions, and have more opportunities to participate in scientific research learning and academic research. It is recommended to “Lead” this group by recognizing them as key advanced practice nursing potential, prioritizing their inclusion in advanced practice training programs [37] and considering them for pilot initiatives such as nurse prescribing rights programs, while also providing platforms for them to spearhead leadership and practice change.

Taken together, these implications offer practical guidance for optimizing specialty nurse development in Beijing. At the same time, because the three‐profile structure was identified among specialty nurses in this highly resource‐rich municipality, its generalizability to lower‐tier cities, rural areas, and the broader national context should be interpreted with caution. Building on these findings, we tentatively propose a “Stretch–Shape–Lead” approach as a theory‐informed direction for tailored interventions. However, this approach has not yet been empirically tested, and long‐term implementation studies are needed before any wider scale‐up.

### 4.2. Demographic Characteristics Associated With Professional Practice Participation of Specialty Nurses in Beijing

The findings indicate that job position significantly predicts the professional practice participation of specialty nurses. Primary nurses responsible for patient care are more likely to be categorized into the Developing Practice Profile. Head nurses and nurses in higher managerial positions were more likely to be classified into the Clinician, Leadership, and Research Profile. This finding suggests that, within our sample of specialty nurses, those in higher positions tended to report higher levels of leadership, education, and scholarly engagement in specialty nursing practice, although not every nurse in this profile necessarily makes a uniformly greater contribution. A higher‐level position generally entails greater responsibility and decision‐making authority. It usually requires assuming more management and leadership functions and playing a more prominent role within the team [38]. Responsible team leaders tend to fall within the Clinician–Educator Profile. They are under the supervision of head nurses and often engage in nursing quality control and team coordination and education demanding strong leadership and instructional skills [39], enabling them to actively contribute to teaching.

Nurses with a Master’s degree or higher are more likely to be part of the Clinician, Leadership, and Research Profile. This implies that a higher education level is connected with engagement in more dimensions of practice [40]. A probable explanation is that postgraduate education can enhance nurses’ knowledge and skills. These include advanced skills such as problem‐solving and critical‐thinking abilities, leadership skills, and the capacity for personal and professional growth [9, 41]. Moreover, due to their research, problem‐solving, and communication skills, nurses with postgraduate degrees are frequently recruited as nursing researchers. As a result, they are more likely to play an active role in nursing scientific research [41]. This indicates that nursing managers should provide good guidance on career development planning and clarify job division to fulll leverage the role of nursing postgraduate students.

Specialty nurses who had held their qualification for 6–10 years are more likely to fall into the group demonstrating advantages in teaching. This implies that nurses of moderate seniority tend to be more proficient in teaching tasks contrary to the results of several other studies. Kodama et al. carried out research indicating that while teaching nurses with 4 years or more of experience exhibit high self‐efficacy when instructing new nurses, particularly in areas such as demonstrating specific methods, enhancing professional self‐growth, and coordinating interpersonal relationships. Experience accumulation, though, does not necessarily translate into greater educational and teaching proficiency [42]. Similarly, Dury et al.’s research revealed that there are substantial differences in the educational and certification standards for specialty nurses across European countries, and interpretations of their roles and capabilities vary widely [43]. Thus, it suggested that the duration since obtaining the certificate might not be the sole determinant of educational and teaching capabilities.

Professional title, working years, and hospital level serve as crucial predictive factors for the professional practice participation of specialty nurses. Nurses with a professional title of senior nurse or lower, with 10 or fewer years of working experience, and those working in first‐ and second‐level hospitals are more likely to fall under the Developing Practice Profile. This, therefore, implies that a high professional title, extended working years, and a high‐level hospital are positive predictors of specialty nurses’ role performance. Specialty nurses with higher professional titles tend to perform their roles more effectively, which aligns with the research findings of Li et al. [34]. This could be due to the different clinical roles assumed by nurses of varying professional titles and the distinct degrees of utilization of specialized positions. The higher the professional title, the more remarkable the nurses’ job‐related competencies are likely to be. With additional responsibilities assigned by hospitals, these nurses are better equipped to fulfill their roles. Years of working experience is significantly associated with nurses’ knowledge and practical skills, especially in the development of expertise within specific domains [44]. As working years increase, nurses accumulate more experience, and their clinical judgment and professional knowledge levels gradually improve. Additionally, the hospital level is correlated with the working environment and resource support available to nurses. Compared to first‐ and second‐level hospitals, third‐level hospitals generally possess more resources and have a more comprehensive management system, offering better development opportunities for specialty nurses [45]. Moreover, higher‐level hospitals often have more intense work environment and complex cases, demanding advanced skills and decision‐making from specialty nurses [46]. This helps them fully use their professional expertise.

Hospitals are advised to create opportunities for experienced, high‐level specialty nurses to showcase their abilities, while also focusing on training and developing junior and mid‐level ones [35]. Simultaneously, the management system for specialty nurses in the hospital should be improved to promote to the optimal level, the roles of specialty nurses.

### 4.3. Limitations

This study has several limitations. First, this sampling was limited to Beijing. Given the higher concentration of medical and educational resources in the city, the findings may not be representative of other provinces and cities in China. Future research should therefore include participants from a broader geographic range. Second, as this was a cross‐sectional study, it does not allow for the determination of causal relationships among variables. Longitudinal studies are recommended to track the progression of specialty nurses’ role fulfillment over time. Additionally, as data collection did not include general nurses, this study does not permit a detailed comparative analysis between specialist and general nurses’ roles. Future research could incorporate the general nurse population, thereby allowing for a more detailed exploration of the distinctions between the two groups in terms of clinical nursing practice, nursing management roles, teaching involvement, and research contributions.

## 5. Conclusion

In summary, the current state of professional practice participation among specialty nurses in Beijing exhibits clear classification characteristics. The optimal three‐profile model consists of the Developing Practice Profile; Clinician–Educator Profile; and Clinician, Leadership, and Research Profile. This study also identified key predictive factors influencing the level of professional practice participation, including job position, educational background, years of specialized qualification, total years of work experience, and hospital level. Nursing managers are encouraged to implement tiered and customized strategies based on the distinct practice characteristics of specialty nurses. Furthermore, we tentatively propose the “Stretch–Shape–Lead” approach that may help optimize the specialty nurse talent structure and support the development of advanced practice nursing in China. As the study sample was drawn from specialty nurses in Beijing, caution is needed when extending the three‐profile structure to the whole country, and future studies should include more diverse regions to validate and refine these profiles.

## Conflicts of Interest

The authors declare no conflicts of interest.

## Author Contributions

Yan Ye: conceptualization, methodology, formal analysis, and writing–original draft. Yinshi Kan: formal analysis and supervision. Tingting Dong: investigation and resources. Jing Zhang: data curation and supervision. Xiaoming Li: data curation and supervision. Lingyu Zhang: writing–original draft. Xuanxuan Fan: writing–original draft. Chunyan Li: resources and funding acquisition. Liwei Jing: project administration, resources, and writing–review and editing.

## Funding

This work received no funding.

## Supporting Information

Additional supporting information can be found online in the Supporting Information section.

## Supporting information


**Supporting Information 1** Supporting File 1: STROBE_checklist_cross‐sectional. In this file, we have checked our manuscript against the STROBE checklist.

**Supporting Information 2** Supporting Table 1: Multicollinearity analysis results of predictors of the professional practice patterns of specialty nurses. In this table, we have performed a collinearity test on the predictors included in the model.

## Data Availability

The data that support the findings of this study are available on request from the corresponding authors. The data are not publicly available due to privacy or ethical restrictions.
