# Supplementary material for: Patterns of Professional Practice Participation: A Latent Profile Analysis of Specialty Nurses in China
Source: J Nurs Manag. 2026 Jan 22;2026:5574431. doi: 10.1155/jonm/5574431 (PMC12824634; doi:10.1155/jonm/5574431)
Supplement: Supplementary file 2 — Supporting Information 2 Supporting Table 1: Multicollinearity analysis results of predictors of the professional practice patterns of specialty nurses. In this table, we have performed a collinearity test on the predictors included in the model. [file JONM-2026-5574431-s002.docx]

**Supplementary Table 1 Multicollinearity analysis results of predictors of the professional practice patterns of specialty nurses**

| **Model** | **Variable** | **tolerance** | **VIF Value** |
| --- | --- | --- | --- |
| 1 | Age | 0.417 | 2.400 |
|  | Level of hospital | 0.984 | 1.016 |
|  | Years of nursing experience | 0.435 | 2.300 |
|  | Years of obtaining the qualification of specialty nurse | 0.787 | 1.271 |
|  | Years of work experience in relevant fields | 0.706 | 1.416 |
|  | Educational background | 0.902 | 1.108 |
|  | Professional title | 0.705 | 1.419 |
|  | Duties | 0.907 | 1.102 |

Notes：Model 1 refers to the multiple regression model that includes these eight variables, which showed significant differences in the univariate analysis results.

Abbreviations:VIF, variance inflation factor
